# Windows of (Dis) Trust: A Situated Psychological Perspective on Understanding the Phenomenon of Trust

**DOI:** 10.1007/s12124-025-09949-w

**Published:** 2025-11-11

**Authors:** Noomi Matthiesen, Paula Cavada-Hrepich

**Affiliations:** Department of Communication and Psychology, Aalborg University, Aalborg, Denmark

**Keywords:** Trust, Distrust, Løgstrup, Spontaneous/sovereign expression of life, Practice theory, Daycare, Parent collaboration

## Abstract

Based on the Danish philosopher Knud Ejlar Løgstrup’s understanding of trust as a spontaneous and sovereign expression of life, we argue that trust is a radically relational phenomenon that manifests itself in a situated manner between people. Distrust arises when our trust is betrayed, either because the other does not live up to our expectations or in some other way raises suspicion that trust may potentially be betrayed. Distrust is thus the negation of trust and requires justification. We argue that trust and distrust are fundamentally different phenomena yet dialectically intertwined. We further argue that both can only be understood relationally and situationally, and thus not as something we carry with us as stable or uniform structures. We analyze examples from the relationship between parents and daycare-professionals, with a particular focus on the windows of daycare centers. Through this, we argue that it is necessary to incorporate a social practice theoretical framework to capture the conditions we have for safeguarding trust and understanding the reasons of why distrust arises. We conclude that Løgstrup’s phenomenological understanding and social practice theory are not immediately commensurable, but both are necessary to develop a comprehensive situated psychological understanding of the phenomenon of trust.

## Introduction

In this article, we will develop a situated psychological approach to the phenomenon of trust. The concept of trust has gained significant attention and discussion in various academic disciplines such as philosophy, psychology, sociology, political science, and economics in the 1980 s and especially the 1990s. The increased focus on the phenomenon is closely linked to the emergence of the post-industrial society, individualism, and technological development that separates people from those who provide a service (Marková et al., 2008). The term “trust” is frequently used in everyday conversation with no ambiguity. There seems to be a taken for granted meaning of trust. However, the concept and theorization of trust within the social sciences is one of the most complex and multidimensional, with a plethora of disciplinary approaches resulting in an incoherent and contradictory field (Wuthnow, 2004). Christensen and Eriksen (2021) helpfully describe three interconnected trends in the research literature on trust: (1) there is a predominance of theories that argue for the fundamental necessity of trust for human survival, (2) many theories understand trust as rational and tied to justifications, resulting in the third trend, (3) a pervasive individualization of the understanding of the phenomenon of trust. They argue, that this stands in stark contrast to the Danish philosopher and theologian Knud Ejler Løgstrup’s understanding of trust as a radically relational phenomenon that is a sovereign and spontaneous expression of life. Løgstrup (1905–1981) studied in the early 1930’s under Martin Heidegger in Freiburg and Hans Lipps in Göttingen, whom he found particularly inspirational. His roots within phenomenology allow for a productive starting point for a situated psychology of trust as it pointedly stresses the situated and relational aspects of human *being*. Løgstrup’s approach to trust thus allows for an investigation of trust that overturns an individualized, cognitive and rational approach to trust and guides our attention to the relational and situated aspects of the phenomenon. In this article, we will therefore take Løgstrup’s understanding of trust as our starting point and argue for the necessity of an understanding of trust as radically relational and contextually situated.

To make the investigation of the phenomenon of trust concrete, we analyze three empirical examples of practices related to windows in daycare institutions and explore how these co-create conditions of sustaining trust between parents and educational professionals. Løgstrup’s exploration of the close connection between trust, ethics, risk, and social expectations becomes particularly relevant in the practice of parent collaboration in daycare settings, where the relationship of trust revolves around the care of small children. The examples are taken from a larger project on parent collaboration in daycare settings. The project followed the educational and care work of pedagogues in eight daycare institutions (with both nurseries and kindergartens) and six childminders in Denmark focusing on the collaboration with parents. The goal was to study the characteristics of this collaboration, in which trust emerged as a foundational aspect. In total, 18 children aged one to five years were followed over six months, where we conducted participant observations and ongoing interviews with both parents, educators, and childminders (For a more detailed description of the project, see: Matthiesen, Tanggaard, and Cavada-Hrepich, 2022). Before we turn to an account of Løgstrup’s theory of trust, we will first argue further for why his theory is a relevant starting point for a situated psychological understanding of the phenomenon.

## Challenging a Decontextualized, Cognitive, and Evaluative Approach

In 1986, the moral philosopher Annette Baier wrote a groundbreaking article on trust and distrust. According to Baier (1986), trust is a relationship where the other is given the responsibility for something one finds valuable and dear. Trust is thus associated with vulnerability and dependence on the other. Baier (1986) defines trust as “accepted vulnerability to another’s possible but not expected ill will (or lack of good will) toward one” (p. 235). According to Baier trust is thus the belief in the others *will* to act well. However, according to Baier, trust should not be understood as an unequivocally positive phenomenon, as it is the condition for deceit and exploitation. I.e. it is only possible to cheat someone, if they trust you. Baier (1986) therefore writes, “Reasonable trust will require good grounds for such confidence in another’s good will, or at least the absence of good grounds for expecting their ill will or indifference” (p. 235). The *assessment* of the other’s motives thus becomes an important aspect of trust. On this basis Baier develops a moral philosophy where it is important to master trust by only showing trust to those who deserve it. This mastery is achieved through reasoning and judgment of when trust is reasonable. Consequently, trust becomes something which one can give or withhold (Christensen & Eriksen, 2021).

It is precisely this evaluative dimension that characterizes much of the literature on trust, with James Coleman’s, 1990 formulation of the rational-choice approach as the most prominent. Many studies investigate how people assess when trust is reasonable and when it would be better to show distrust. These theories of trust therefore deal with risk calculations, rationality, assessment of credibility, etc. (see for example O’Neil, 2002 and 2018; Hardin, 2006; Lahno, 2004). Trust becomes a rational and cognitive phenomenon that we can master at will, based on more or less well-founded calculations of risk. Furthermore, akin to dominant evolutionary perspectives, Baier (1986) describes how the child who shows trust in the food the mother gives her has a greater chance of survival than the child who is inclined to show distrust. These evolutionary theories link the ability to show trust in each other to human historical survival, arguing that trust is a prerequisite for cooperation between people (see, e.g. Ostrom and Walker (2003). Without claiming further similarities, Niklas Luhmann’s (1968/1999) understanding of trust is likewise linked to a logic of necessity and survival. He developed a functionalist approach to trust, where he argues that trust works to ensure complexity-reduction. For Luhmann, the fundamental challenge of social life is the fact that other people have the freedom to act in ways that differ from the ways in which I act, and from what I have come to anticipate as expected behavior (Mortensen, 1999). Trust, according to Luhmann, is the phenomenon that enables us to reduce this complexity of difference, which is necessary for maintaining social order. Trust thus becomes a necessary condition for social survival. Christensen and Eriksen (2021) argue that these theories are all thus based on an *individualized* understanding of trust, arising out of *functional* interests, and as something a person carries with them as a *stable* characteristic or ability that they can show or withdraw at will based on *rational* judgements. This approach to trust stands in stark contrast to Løgstrup’s radically relational understanding of trust as a sovereign expression of life that is *given* to us relationally. His theory describes trust as something that is neither conditional nor can be mastered. Situated psychology examines psychological phenomena as something that arises in specific situations in a relational manner, and Løgstrup’s theory is therefore a suitable starting point for understanding trust as radically relational, rather than as an individual quality that can be refined and mastered. This is what we will now turn to.

## Trust and the Ethical Demand

In “The Ethical Demand,” Løgstrup (1956/2020) describes how, in all human interaction, we are exposed to each other: “… we *are* each other’s world and each other’s fate” (p. 16). His starting point is that every interaction with the other is, to some extent, vulnerable as it involves self-exposure. The encounter with the other presupposes a willingness to step forward in vulnerability, trusting that the other will take care of our fragility. Stern (2019) describes it as follows: “When I trust someone, I am delivered up to the other person who may then decide what to do with this vulnerability — to respect it or abuse it” (p. 11). Whilst he shares this point of departure with Baier, they draw very different conclusions from this analysis of the relationship between vulnerability and trust. For Baier trust is conditional and requires judgment in order to protect one’s vulnerability. For Løgstrup, trust is unconditional and spontaneous and because of our dependence on one another we are required to act well in the face of the other’s vulnerability. The central point is that we spontaneously trust one another to take care of our vulnerability, which is the basis of ethics. Thus the fundamental premise of the ethical demand is to always do what is best for the other. A demand that, according to Løgstrup, is radical and unfulfillable.

The ethical demand is radical in the sense that it requires us to unconditionally do what is good for the other, regardless of whether the other is a close relation or a stranger. It is also radical in its unfulfillability, as one inevitably puts oneself first when trying to fulfill it. When my child jumps off a table trusting that I will catch her, I catch her because it is what is needed in the situation, not because it makes me a good parent, or because I believe she will love me more if I do so, nor because I consciously strive to fulfill an ethical demand. I am not trustworthy for the sake of trustworthiness. I do not perform in accordance with social norms because it is expected of me to be considered a trustworthy mother, but because it is the right thing to do in the given situation for my child (Faulkner, 2017). The demand is thus not impossible to fulfill, but becomes unfulfillable as soon as we consciously strive to fulfill it for its own sake.

Through the analysis of the ethical demand that arises from our trust in each other, Løgstrup (2020) shows that trust is a prerequisite for life itself: “We simply could not live, our life would wither away and become stunted, if we were in advance to meet each other in distrust, or assume that the other person is stealing and lying, dissembling and leading us on” (p. 9). Trust is thus fundamental to our existence. This does not mean, of course, that we *always* meet each other with trust, only that trust is foundational in human relations. The first chapter of “The Ethical Demand” opens with the sentence: “It is integral to human life that we normally meet each other with natural trust. This happens not only when we meet a human being we know well, but it also holds when we meet a complete stranger. Only in special circumstances do we face a stranger with distrust in advance” (Løgstrup, 2020, p. 9). His point is that trust is *immediate* rather than based on judgements. When we ask the stranger what the time is, we do not think the other is lying when they respond; when we are offered a cup of coffee, we do not think it is poisoned; when we pass someone on the street, we do not think they will trip us. In our study of trust in daycares, a mother said the following in an interview:

Dorthe: Laila [the childminder] answers honestly, … I assume (laughs). Or, at least, I can’t imagine otherwise.

She is describing her collaboration with the childminder, Laila, and reflects on Laila’s answer to the question at the end of the day about how the day has been. Only after she has said that Laila answers honestly, does it occur to her that she does not *know* whether this is the case, because she has no basis for knowing this. And she reflects further that she cannot imagine that Laila is not telling the truth. Trust is thus immediate. Distrust, on the other hand, arises when there is reason for it. We become suspicious when we realize the other has lied, or when the other behaves in a way that gives rise to our distrust. Distrust requires justification. It arises when our trust is betrayed, or we have learnt that it is right and proper to have distrust in a given situation (Løgstrup, 2020).

For Løgstrup, trust is thus primary while distrust is the negation of trust (Løgstrup, 1961). This does not mean that trust, according to Løgstrup, is unequivocally positive. There may be situations where it is ethically right not to show trust. However, trust is, according to Løgstrup, fundamentally a positive phenomenon necessary for our basic way of life and can at the same time be negative in specific situations (Nierker, 2017). It is a question of rank: “The difference for me between first and last is as much a matter of rank […] When I say that trust is primary, I mean that distrust is the negation of trust and as such founded in trust” (Løgstrup, 1961, p. 229, our translation). This is because trust is a spontaneous/sovereign expression of life, whilst distrust requires justification. We will now turn to a description of what is meant by a spontaneous and sovereign expression of life.

## Trust as a Spontaneous and Sovereign Expression of Life

Central to Løgstrup’s understanding of the human being are the spontaneous or sovereign expressions of life, where the phenomenon of trust is the paradigmatic case (Read, 2019). He developed the concept of expressions of life throughout his authorship, especially in the latter part, and uses “spontaneous” and “sovereign” interchangeably, without any particular distinction between them. He primarily uses the term “sovereign expression of life” in the book “Controverting Kierkegaard,” where he describes expressions of life such as trust, openness of speech, and compassion. From his early writings as a parish minister, he describes how adults (in contrast to the child) strive to be sovereign over their own lives, i.e., that the adult considers himself and his world as something within his own control. As Bugge (2014) writes, “the adult can repress that he and his world have a limit” (p. 70, our translation). Instead, he describes sovereign expressions of life as phenomena that sustain our existence, but which we have not created ourselves. These are phenomena that do not arise by virtue of our own efforts (Olsen, 2015). This means that we cannot master trust or cultivate it. Instead, one can only safeguard and protect it, in the same way that one can safeguard love and act in accordance with it (Løgstrup, 1972).

It is in the later untranslated publication “Norm and Spontaneity” that he unfolds the spontaneous nature of expressions of life. The word “spontaneous” etymologically means “unforced and without ulterior motives.” These are phenomena that exclude any control, assessment and calculation: “The sovereign expression of life comes ahead of us, we are seized by it. That is where spontaneity lies” (1972, p. 17, our translation). It is in the relationship and in the presence of the other that the expressions of life make demands on us (Løgstrup, 1972, 2013). The “forced” or “encircling” phenomena (e.g., offense, jealousy, and envy) direct us inward and circle around the self. The sovereign expressions of life move us out of ourselves and towards the other. Løgstrup insists that we realize ourselves in concrete actions in the fulfillment of the sovereign and spontaneous expressions of life. Humans are bound to the world through sovereign expressions of life such as trust, mercy, and openness of speech. It is precisely this binding to others, and not the effort to break it, that enables the realization of the self (Løgstrup, 2013).

## Expressions of Life as Pre-Cultural?

Løgstrup distinguishes between ‘ethics’ and ‘morality,’ which etymologically speaking are the same. ‘Ethics’ comes from the Greek ethikós, meaning ‘manner’ or ‘custom,’ while ‘morality’ comes from the Latin moralitas, meaning ‘custom.’ Both words etymologically denote common viewpoints on how one *should* act (Brinkmann, 2016). However, Løgstrup consistently uses the term ‘ethics’ to denote a universally given ethical demand to do what is good for the other, while ‘morality’ denotes the cultural norms and conventions for how one should act in a given situation. The sovereign expressions of life, like the ethical demand, are according to Løgstrup *pre-cultural* phenomena. This means that they arise from the fundamental condition that we are relational beings. Both the ethical demand and the sovereign expressions of life are thus universal in the sense that they are not tied to specific cultures, specific social forms, or specific historical movements, but rather to the condition that humans are always and in all circumstances in relation to other people (Nierker, 2017). However, this does not mean that they are abstract or manifest themselves disconnected from the moral order. The always arise in the concrete relation to the other.

The idea that expressions of life, such as the phenomenon of trust, should be pre-cultural has met much criticism. Bauman (2003) comments with a touch of sarcasm, that the idea for the book “The Ethical Demand” was conceived while Løgstrup was a parish priest on the rather idyllic island of Funen before World War II, and Bauman proclaims, “With due respect to the friendly and sociable residents of Aarhus where Løgstrup was to spend the rest of his life teaching theology in the local university, I doubt whether such ideas could gestate in his mind once he had settled in that town and faced point-blank the realities of the world at war and under occupation, as an active member of the Danish resistance” (Bauman, 2003, p. 87). Sløk (1979) similarly criticized Løgstrup’s concept of expressions of life as pre-cultural, pointing to the obvious fact that the world does not seem to be characterized by trust, mercy, care, etc., so claiming these as primary and pre-culture seems absurd.

We find an example of deep and pervasive distrust in Umbres’ (2022) ethnography of a post-communist village in Romania. Here, society is characterized by trust in family members and deep distrust of everyone else in the village. The distrust is so pervasive that the village members chose, after the end of communism, to destroy two large newly built communal agricultural buildings and carry the almost unusable remains home to their own plots of land, rather than continue the cooperation by sharing the functional large buildings. Distrust is primary in this community. Løgstrup, however, argues that culture, the local moral order, and social conventions create conditions for the sustaining sovereign/spontaneous expressions of life: “… distrust fills at least as much in our existence as trust, perhaps even much more” (Løgstrup, 1961, p. 231, our translation). Culture and norms create conditions for whether distrust stifles trust. The villagers in the Romanian village have learned through the oppressive communist regime to distrust not only the government but also the local community. Løgstrup’s point is that we learn to distrust when it is necessary. Distrust can in a particular culture or community become dominant, when there are reasons for this. However, this is not the same as saying that distrust is primary, as it always exists as the negation of trust. One has learned to distrust, and it can be justified with reference to personal experiences or cultural narratives. Trust neither can nor should be justified, but distrust can be explained by referring to experiences, common narratives, or social norms. Thus, trust still takes precedence even when distrust is most prevalent (Løgstrup, 1961).

However, whilst we agree that trust is primary, i.e. we trust the other without reason as long as these conditions are present, the notion of trust as precultural only makes sense if we limit our understanding of trust to a very basic form consisting of the spontaneous, precognitive willingness to be vulnerable in the encounter with the other. But looking closer at the phenomenon, we quickly see that both the content and the form of trust are highly cultural. In other words, the content and form are situated and connected to the particular normative practice in which it arises. We will now turn our attention to this cultural and normative aspect of trust.

## The Relationship Between a Universal Expression of Life and a Situated Morality

While the ethical demand and expressions of life according to Løgstrup are universal, they are by no means abstract or decontextualized. They are instead situated, as the demand and expressions of life arise in the encounter with the concrete embodied other. They arise from the *microsocial* condition that we are placed in relation to each other. Social conventions, the moral order, and legal laws, on the other hand, arise from *macrosocial* conditions, where we collectively must agree on what is right and reasonable in given circumstances (Fink, 2017, p. 72). Every social encounter will, however, contain both forms of normativity. The relationship between the different forms of normativity tied to the universal expressions of life and the local social order becomes clearer when we look more closely at the relationship between trust and distrust. Here, Løgstrup’s (2020) concept of social conventions is useful for our inquiry into the constitutive aspects of trust. He describes social conventions as social agreements that “ease our social interaction with one another, making it smooth and effortless, especially by saving us from laying our souls bare. Without the protection of conventional norms, any social intercourse with other people would become unbearable” (p. 18). Social conventions function as protection so that we do not stand unmediated before each other in total vulnerability. They give *content* and *form* to trust. While trust is a sovereign expression of life that arises spontaneously in the encounter with the other, the content of trust is culturally given by a local and situated morality. This means that the expectations of *what* the other should do and how they do it to live up to this are given by social conventions, norms, laws, etc. The *form* of trust, i.e. how it is expressed and understood by others, is likewise given culturally by the social conventions (see further Matthiesen et al., 2022).

Sometimes our trust is simply tied to a basic belief that the other means us well and thus will not humiliate or ridicule us. Other times, the content of our expectations is more complex. When we drop off our child at a nursery, there is an expectation that the pedagogue will care for our child both practically (helping the child with the toilet, providing food, ensuring they stay warm) and in a way that ensures the child’s development (through bodily contact, encouraging independence, language stimulation, etc.). In our study, a father talked about repeated betrayals of his expectations that resulted in distrust and eventually finding another daycare for his children. For instance, he described an experience of picking up his two children in freezing weather on the playground without them being properly dressed. Or an episode where the daughter had been sitting on the toilet for a long time, calling for an adult without being heard, resulting in her still sitting there, covered in feces, when the parents picked her up. This was a violation of his trust in the pedagogues taking care of his children in accordance with the local societal norms and conventions. The father said that even when everything looked fine when picking up and dropping off, he and his wife met the situation with suspicion:

Thomas: And if it’s already like that, that you don’t really know how it really is, or that it’s a bit tense, then it’s very easy to think that the atmosphere is not good when you’re not there. That the positive is a scene made for the parents.

Even the situations that met the expectations were now understood as an inauthentic performance. The example shows that trust and distrust are closely linked to the social conventions of practice and the associated expectations.

In Løgstrup’s work with social conventions, it is not entirely clear how these are produced and reproduced, and what they mean for the possibility of safeguarding trust. In a discussion about the relationship between trust and distrust, Løgstrup (1961) asks the question, “Is there a philosophical psychology alongside the scientific one? I believe there is” (p. 230). By philosophical psychology, he means the investigation of the foundations of a particular phenomenon (i.e. trust) and how the phenomenon is experienced by virtue of our humanity. He describes the “scientific psychology” as a “status rapport psychology” which aims at an analysis of how a phenomenon (in this case trust) currently stands in everyday life and conditions for this status in social practices. However, the relationship between this psychological status report and the philosophical psychology remains somewhat unclear. We will therefore in the following try to exemplify this relationship through an analysis of three different concrete windows in the daycare, all of which have significance for trust. The windows as material artifacts play a significant (though often overlooked) role in the emergence and maintenance of trust in the practice of collaboration of care in daycare. We will argue that incorporating concepts from practice theory can help us grasp trust as an empirical phenomenon that manifests itself relationally, or alternatively is displaced by distrust, in a social normative order. Practice theory (as described in Birk et al., 2025) can thus help us develop a “psychological status report” whilst insisting on a dialectical relationship between the “philosophical” and the “status rapport” psychology. In other words, practice theory helps us see the conditions and current possibilities of (dis)trust in a concrete and situated practice. We will combine this approach with Løgstrup’s understanding of trust as a spontaneous and sovereign expression of life to formulate some central points for further work on developing a situated psychology.

### The Waving Window

In daycare institutions, there are often rituals associated with saying goodbye to mom or dad when the child is dropped off. In some places, the child is encouraged to start playing with another child or an adult before the parent leaves. In many institutions, there is a practice of waving at a window together with the pedagogue/childminder. These windows are routinely called “waving windows.” In our study, there was a childcare center with a “waving window” by the front door. It was a floor-to-ceiling window with a large wooden horse placed by it. Here, the parent says goodbye and then leaves through the door, after which the pedagogue helps the child onto the horse. The parent can turn around when they are near the parking lot and wave to the child, who sits and waves back on the horse with the pedagogue by their side, perhaps with their arm around them. It is a very clear choreography that the parent, child, and pedagogue perform together. The choreography is associated with repetition, security, and clear expectations for all involved.

The window creates specific conditions for maintaining trust or for the emergence of distrust. To understand the significance of the window, the ritualized actions, the physical contact between the pedagogue/childminder and the child, as well as the parents’ actions, we must understand their conditions of participation in the historically and politically produced practice (Dreier, 2015). Løgstrup uses the term “social conventions” to describe the normative expectations that exist in specific contexts, but he does not offer concepts to unfold this normative order, how it arises, and what significance it has for the actors’ possibilities of participation. The relationship between the spontaneous expressions of life and the local and situated normativity thus remains unclear. Here, we must instead draw concepts from social practice theory.

Social practice theorist McDermott (1977) describes trust as a relational phenomenon characterized by “a crucial subset of the working agreements people use to make sense of one another” (p. 199). He thus connects trust with the subtle and often pre-reflective expectations people have of each other in various practices. This corresponds largely to Løgstrup’s (2020) description of the local moral order and social conventions, as described above. They can also agree on the relational nature of trust. McDermott (1977) writes:

“It is important that the reader not take “trusting relations” in the ordinary sense, which implies that trust is a property of a person’s personality. The developmental literature is filled with references to a child’s acquisition of “basic trust” as if it becomes a property of the child to be used in all situations. However, I am talking about trust as a quality of the relations among people, as a product of the work they do to achieve a shared focus. Trust is achieved and managed through interaction” (p. 200).

While McDermott and Løgstrup can agree that trust is a phenomenon that arises between people, Løgstrup would disagree with the last sentence in the above quote. According to Løgstrup trust is not something that can be “achieved” or “managed” as trust arises spontaneously. Instead, trust can be protected and maintained, whilst *distrust* is negotiated, managed, and perhaps pushed aside, allowing for the emergence of spontaneous trust. We will expand on the relationship between trust and distrust below, in the analysis of the vestibule window. For now, we will stress another important point: it is the normative order of social practice that creates the conditions for the emergence and maintenance of (dis)trust. Trust is thus closely connected to expectations of how one *ought* to act. However, although trust arises in a social practice, it is not in itself a social practice. Christensen and Eriksen (2021) describe it as follows: “Moreover, it [trust] is not a social practice that can be established (like money or marriage); rather, basic trust is an empirical fact about human life which is connected to the fact that we are delivered up to others and thus interdependent and vulnerable, and which may be promoted or undermined, but never eliminated as a condition of human life” (p. 36). However, it is the concrete social practice, i.e., the relationships, materiality, norms, etc., that create the conditions for *safeguarding* trust and for the possible emergence of distrust.

The content and form of trust, what we trust the other to do, and the conditions for safeguarding it, are tied to the practice. In social practice theory, everyday life is understood as socially (re)produced in historical and political practices (Lave, 2011). These are established, maintained, and changed through human participation based on their position and the possibilities for legitimate participation available in the practice. People have intentions with each other in these practices, where they are directed towards specific concerns (see Birk et al., 2025 for a more detailed elaboration). Specifically, in the Danish context the drop-off practice in daycare involves transferring the responsibility for the care of the young child from one adult (the parent) to another (the educator/childminder) for a several hours each day. Parents are expected to say goodbye without prolonging the drop-off. They are expected to smile and wave unperturbed, regardless of how the child reacts, to signal to the child that everything is fine and safe. It would be entirely illegitimate in the practice for the parent to break down crying or regret and run back to the child. Such behavior would undermine the pedagogues trust in the parents, and a budding distrust would likely arise. While parents are expected to leave the practice with an appropriate balance between calm and haste, the pedagogue is expected to act as an unwavering caregiver. She is expected to show closeness by holding the child; show interest in the child; comfort the child if they cry; help the child engage in play, etc. The drop-off practice is thus permeated with expectations for specific actions that demonstrate a transfer and the taking up of responsibility for the child’s care.

While Løgstrup and Baier do not agree on the essence of trust as a phenomenon, they agree that trust is closely connected to vulnerability, what we hold dear and thus to an expectation of care (whether it is our own existence or our children, as in the present analysis). Baier (1986) describes it as follows: “Kant’s neighbors who counted on his regular habits as a clock for their own less automatically regular ones might be disappointed with him if he slept in one day, but not let down by him, let alone had their trust betrayed. When I trust another, I depend on her good will toward me” (p. 235). Trust is thus not tied to expectations of mechanical and causal relationships. If the child at the waving window got their finger caught on the way up onto the waving horse, it is not a breach of trust. It would become be a breach of trust if the child’s finger was caught due to negligence by the pedagogue, or if they ignored the child’s crying and continued waving smilingly out the window while the child sobbed. It is thus a breach of trust when the pedagogue does not provide care in accordance with the normative order, but not when an accident occurs. Marková et al. (2008) argue that the word “comfort” (in Danish “trøst”) in the Nordic languages has the same etymological origin as the word “trust” in English. Both words originate from the Old Norse word “traust,” which means “help, protection, support.” Trust and comfort are intertwined and mutual phenomena. It is precisely this normative order with expectations of each other that allows us to hold each other accountable when one does not meet the expectations of good and appropriate behavior (McDermott, 1977).

The waving window enables a ritualized practice and transparent customs for parents, educational professionals, and children, ensuring that there are reasonably clear expectations of each other, and thus good conditions for safeguarding trust. The window also allows a view into the practice from a distance. Although the parents have left the childcare center and are physically separated from the child, the window allows a glimpse of how the child reacted to the farewell and how the professional provides care in the situation, sustaining trust or potentially giving rise to distrust. Another similar materially supported trust-preserving practice is that some educators/childminders send a message or a picture of the happy child when the child is no longer upset about the farewell. The material conditions in the practice can thus help create the conditions for maintaining trust, but they can also challenge it. We will look more closely at this in the following example.

### The Vestibule Window

In one of the study’s daycare institutions, there was a vestibule by the front door. In this vestibule, the cloakroom was on the right-hand side, the staff room on the left, and the entrance to the large common room was straight ahead. The entrance area was made of glass, so you could see into the vestibule when approaching from the parking lot. There was also a glass door and glass wall into the common room. This meant you could get a sense of who was in the common room as soon as you arrived, and the pedagogue could see that someone was entering. The glass door kept the cold from the front door out of the common room. At first sight it was an ingenious architectural design. But the common room was large and had high ceilings, which resulted in a high level of noise, drowning out the sound of someone arriving. Additionally, the table where the pedagogues sat during morning drop-off was far from the vestibule, making it difficult to see the arrival of parent and child. Often, the kindergarten children would go out to the vestibule to say goodbye to their parent. However, the pedagogues had to stay at the large common table, as there were many other children in the area, and the smallest nursery children often sat on their lap. Usually, the kindergarten child would happily run back after saying goodbye, but sometimes they would get upset as they watched their parent drive away. In such cases they sat alone crying in the vestibule, and the pedagogues, who sat far away at the common table in a noisy common room, couldn’t hear them. This meant that sometimes parents would arrive at the daycare institute and the first person they met was a very unhappy little person sitting in the vestibule. A mother described this in an interview:

Andrea: This layout is not very good because there is a long way from the common room to the exit. For example, if they are in the vestibule, and the child is really upset because the parents have left, the adults can’t hear the child crying. I find that difficult. It happens maybe once every two weeks. So, I’ve started saying: “You know what, I know Gertrud is right over there. Let’s go to her. Because I also understand that the adults can’t be everywhere.

Andrea describes how once every two weeks she arrives at the daycare, and the first thing she encounters is a crying child. When you enter the building, the first impression in such cases will be that there are adults who are not providing the expected care in the transition from home to daycare. But the mother reflects on the conditions for providing care. In her statement, she shows that she still trusts the adults as caregivers. She helps the child to Gertrud precisely because she does not question that Gertrud both *wants* to and *can* provide the necessary care. Her trust is still spontaneous and unconditional. However, Andrea reflects on the fact that the adults have poor conditions for providing care. In this specific case, she points to the material conditions of the practice that make it difficult for the educators to notice the child’s need for care. In other parts of our empirical material, parents similarly reflect on the pedagogues’ conditions, either their general low level of staffing, the external political demands for learning goals, or the daycare’s management. The parents unanimously said that they trusted the individual pedagogue but could express concern about the pedagogues’ conditions for meeting the expectations of care (see Matthiesen et al., 2022).

The example helps us better grasp the phenomenon of trust in two ways. First and foremost, there is the somewhat banal point that the political, material, and social conditions in the practice all have significance for whether the pedagogues can meet the expectations (Lave, 2011). If trust is a spontaneous expression of life, it is not conditional on participation. But the particular conditions in the normative moral order, are significant for the pedagogues’ and parents’ ability to safeguard trust and protect it from the emergence of distrust. The second important point the example points towards is that Andrea shows two forms of trust. She shows a basic spontaneous trust in educator Gertrud. She has no doubt that if Gertrud knew the child needed care, she would want to help and do so competently. But she also shows a more reflective, calculating, and demanded trust. She asks the question of how she can understand the situation with the crying child in the vestibule. And she reflects on the significance of the daycare’s architecture and the conditions for care. She concludes that she trusts the pedagogues despite these conditions, as long as the others (i.e., the parents) lend a hand and help the children find a relevant caregiver. This is an example of complex and demanded trust (Løgstrup, 1961; Christensen & Eriksen, 2021).

Not all trust is, therefore, spontaneous. Complex trust is based on a more or less conscious reflection, where it is assessed as distrust emerges. The assessment is upon whether the distrust is justified, that is, whether the other does not live up to the expectations in the social normative order. Trust and distrust are here dialectically intertwined, with distrust lurking as a backdrop often easily emerging and displacing trust. Spontaneous trust is a fragile expression of life that can easily be stifled. As Nierker (2017) writes: “The expressions of life are too fragile to prevent collapse, and they are too indispensable to be wiped out” (p. 206). Doubt, or distrust, can show itself in cracks when others do not live up to our expectations. In such cases, one has the opportunity to either give in to distrust or hold on to doubt and investigate it. Distrust can be dismissed, and we can strive for a complex trust. Striving for trust requires interrogating one’s distrust, displacing it and acting as though one has trust. This allows for the possible reemergence of basic trust that once again can be spontaneous. Alternatively, distrust can take hold, and the sovereign expression of life is displaced. Once distrust has displaced trust, it can be difficult to create space for trust to show itself again (Nierker, 2017). This also means that one can trust that the person can handle a specific task (i.e., live up to the expectations in the normative order) but at the same time not trust their goodwill or integrity (Marková et al., 2008). Through participation in practices, we *learn* what we should distrust and when it is appropriate to be on guard. How one makes this type of assessment is a complex task to describe and goes beyond the scope of this article. But the assessment is often pre-reflective and closely intertwined with overarching societal narratives. We will illustrate this with yet another window from the daycare.

### Window into the Changing Room

The changing room in nurseries is a space associated with intimate closeness. On the changing table, the three-year old Alma is lying on her back and laughing while the pedagogue Beatrice changes her diaper. They talk about the pictures of animals next to the changing table. Alma points, and Beatrice says, “Chicken.” “Chi’ken,” replies Alma. “Peep peep,” says Beatrice as she fastens the clean diaper, and Alma responds, “Peep peep.” Beatrice tickles her feet a little and takes her time pulling up her tights while Alma points to a picture of a duck. The conversation repeats itself. “Shall we go join the others?” asks Beatrice, and Alma nods. She gets to press the button on the adjustable table, so it lowers, and she can climb down and run out to the others.

There is a material reality that insists that changing a diaper is done in a hygienic place away from the other children and the eating area. It is also a task that does not tolerate many interruptions, as anyone who has changed a diaper knows; once it is opened, the task must be completed with a certain efficiency. The practice of changing a diaper in the daycare is therefore characterized by closeness, calm, and one-on-one contact without interruptions. Changing a diaper is a practical measure, but it is also very much a practice of care.

However, the changing room is also characterized by being a very intimate space, and thus it also carries a grave risk. Sexual abuse of young children in daycare is fortunately very rare, but it is among the most horrifying and destructive violations of trust and of life itself. For this reason, in all the childcare centers where we conducted fieldwork, there was a window into the changing room where the changing table is visible to those in the adjacent rooms. The window thus provides access to a care practice that is associated with risk. Trust is associated with situations where we depend on another. It is thus linked to the dependency between people, but where there is a risk that that this dependence can be betrayed. As Zittoun (2014) writes:

“Trust demands a form of risk-taking; the person will only know in the future whether that trust was rightly or wrongly placed in a person or a situation. Trust is hence situated in time; its confirmation always lies in the future” (p. 128).

It is precisely the aspect of the future-orientedness of trust that makes trust interconnected to risk. The significance of *time* can be described as the difference between familiarity and trust: “Familiarity is tied to the past; trust and distrust to the future” (Mortensen, 1999, p. 18). When one drops off their child at daycare, the aspect of lacking knowledge becomes very relevant. The parents do not know if what they see while they are at the daycare is actually what happens when they are no longer there. The separation involves a lack of knowledge and thus also a risk that the child does not receive the care the parents expect. That point that trust is connected to risk directs our attention once more to the ever-lurking backdrop of distrust that can displace trust as soon as our attention is drawn to the risks entailed in the social practice.

Risk and doubt emerge at specific times when there is reason for it. When one looks through the window into the changing room, one sees a practice of care unfolding. The pedagogue Beatrice and the child Alma are in a close and developing interaction. If one fleetingly becomes aware of the window, which one had just (unaware) looked through, one becomes reminded of the immense risk that the practice carries. The window is there because we, as a society, find it important to protect our children from abuse and neglect, and the intimate changing room carries this risk. But the window also contains a promise of transparency and thus a form of assurance of the social contract of care in the normative order. The window contains a double message: the changing practice carries a terrible risk, but transparency offers a form of sporadic knowledge about what is happening in the room, thereby reducing the risk. It is likely that the spectator, observing the interaction between Beatrice and Alma will soon lose sight of the window again, and instead merely look through it rather than at it. The fleeting distrust is displaced by spontaneous trust as the interaction between child and pedagogue is in accordance with the normative social order. But perhaps Beatrice does something unexpected that does not align with our expectations, and distrust emerges. Or perhaps we have just read a newspaper article about a case of abuse in another kindergarten. The media’s accelerated information distribution also increases the emergence of distrust. Or what if Beatrice is not named Beatrice, but instead named Bernhard? The culturally created gendered understandings of who commits abuse also influence whether distrust displaces trust. There is a multitude of societal narratives and personal experiences, all of which influence what awakens our distrust and how it can be justified or challenged.

The window into the changing room helps us grasp that trust is always embedded in a larger societal context and that the individual’s own experiences and history matter. We also become aware of the fragility of trust. Even though trust is an immediate and basic expression of life, tied to the fact that humans are relational, it can be displaced by betrayal, or the risk and concern for betrayal that embeds itself in practices and societal narratives.

## The Significance for the Development of a Situated Psychology

“Sovereign expressions of life are basic ways of being in the world that we as human beings have not authored ourselves, neither as individuals nor as a community. They are not learned, chosen, or willed by us, but they express the form of life of beings such as ourselves. To be a human being is, among other things, to show basic trust” (Christensen & Eriksen, 2021, p. 32).

A situated psychology must have as its central concern the question of what it means to be human in a relational and material world. In this article, we have described trust as a phenomenon that is a central part of being human. We have described it as a phenomenon that cannot be owned by the individual and cannot be summoned or controlled. Instead, it is a radically relational phenomenon that arises in the encounter between people and stems from the fact that we constitute each other’s fates.

Løgstrup is not concerned with delineating a psychological status report of trust. Instead, he is “concerned with determining what life is aimed at, insofar as it concerns the good or moral life” (Pahuus, 1994, p. 42, our translation). We have argued that social practice theory provides us with helpful understandings to delineate the conditions of local practices for safeguarding trust and the reasons for the emergence of distrust. Social practice theory can thus supplement Løgstrup’s analysis of the spontaneous/sovereign expressions of life to gain an understanding of both basic and complex trust and the relationship between these (Christensen & Eriksen, 2021).

The spontaneous expressions of life are not directly connected to or arise from the local moral order, as they stem from fundamental conditions related to parts of existence that are given by virtue of human interdependence. But the *content* and *form* of the expressions of life arises from the concrete practices: “The content of the sovereign expression of life is given by the situation and the relationship to the other person, that is, by my perception of the situation and the relationship, their current circumstances, and history” (Løgstrup, 1968/1994, pp. 94–95, our translation). It is when people act in accordance with the expectations in the normative social order that trust can arise as a spontaneous expression of life. Or rather, when people do not act in accordance with the normative social order, the possibility of trust is stifled, either through fleeting suspicion, uncertainty, or actual distrust, which they can justify.

Fink (2017) argues that the ethical demand is a form of normativity that is incommensurable with a normativity found in social conventions, but that the two normativities are complementary. He uses the concept of “complementarity” (p. 69) to describe the relationship between the ethical demand and other moral demands, and we will here extend his argument to also apply to the relationship between the sovereign/spontaneous expressions of life and the conventions in the local moral order. The concept of “complementarity” is borrowed by Fink (2017) from Niels Bohr, who in October 1954 (at the same time Løgstrup was completing work on “The Ethical Demand”), gave a lecture at Columbia University titled “Unity of Knowledge.” In this lecture, Bohr described the development of physics in the early twentieth century and how quantum physics behaved quite incomprehensibly when interpreted through classical physics. He argued that the logics that apply in quantum physics and classical physics are fundamentally different but simultaneously complementary. This applies, according to Bohr, to all forms of science, including psychology (Fink, 2017)^1^.

The world consists of logics that are incommensurable but simultaneously necessary to understand what it means to be human. Trust is a spontaneous and sovereign expression of life that is radically relational and situated, due to the fact that we are beings dependent on each other. When we drop off our children at daycare, trust is spontaneous. We have not agreed that it should arise to facilitate our cooperation functionally, and we cannot decide to have trust and control it. But the practice, its historical and political development, the material conditions, the normative order and associated expectations of appropriate behavior, as well as the participation of the actors in the practice all have significance for the content of trust and the conditions for safeguarding it. Social practice theory struggles to describe what trust fundamentally *is* and what normativity arises from the condition that we trust one another. On the other hand, we need practice theory to describe the local normativity of the practice, and the situated conditions related to the practice’s purpose, materiality, power structure, participation of others, etc. It is our hope to point out the necessity for situated psychology to develop an analytical conceptual apparatus that can capture the situated existential aspects of humanity that are beyond our own control and related to the fact that we exist in the world as relational beings dependent on each other. And also, at the same time, develop concepts that can analyze how these aspects are manifested in lived lives in locally situated practices.

## Data Availability

No datasets were generated or analysed during the current study.

## Footnotes

1 The logic of complementarity has been thoroughly discussed in relation to cultural psychology in the Niels Bohr Lectures on Cultural Psychology. See for instance Valsiner (2018).
